# Septic Malleolar Bursitis in a Patient with an Ankle Electronic Monitoring Device: A Case Report

**DOI:** 10.5811/cpcem.2020.12.50299

**Published:** 2021-01-16

**Authors:** Bart Besinger, Sydney Ryckman

**Affiliations:** Indiana University School of Medicine, Department of Emergency Medicine, Indianapolis, Indiana

**Keywords:** bursitis, ultrasonography, electronic monitoring device, ankle joint, case report

## Abstract

**Introduction:**

Septic malleolar bursitis is a rare cause of ankle pain and swelling. It has been described in certain occupational and recreational activities that involve tight-fitting boots, such as figure skating. Court-ordered electronic monitoring devices are often worn on the ankle. It is not known whether these devices are a risk factor for the development of malleolar bursitis.

**Case Report:**

We describe a 41-year-old male under house arrest with an electronic monitoring device on his right ankle who presented to our emergency department with several days of progressive pain and swelling over the medial malleolus. Point-of-care ultrasound revealed a thick-walled cystic structure consistent with medial malleolar bursitis. Bursal aspiration was performed. Fluid culture yielded *Staphylococcus aureus*.

**Discussion:**

Emergency physicians regularly see patients with ankle pain and swelling and must consider a comprehensive differential diagnosis. Septic malleolar bursitis is an uncommon but important cause of ankle pain and swelling that requires prompt diagnosis and intervention. Point-of-care ultrasonography may aid in the diagnosis. Additionally, emergency physicians should be aware of potential complications related to electronic monitoring devices.

## INTRODUCTION

Malleolar bursitis is a rare cause of ankle pain and swelling. In many cases, it can be attributed to ill-fitting footwear or repetitive trauma from occupational or recreational activities such as figure skating. It is unknown whether court-ordered ankle electronic monitoring devices contribute to the development of ankle pathology, including malleolar bursitis. We report a case of a 41-year-old male who developed right medial ankle pain and swelling while wearing such a device. His physical examination was concerning for septic medial malleolar bursitis. This was confirmed with point-of-care ultrasonography and aspiration and culture of bursal fluid. We believe this is the first reported case of malleolar bursitis associated with the use of an electronic monitoring device.

## CASE REPORT

A 41-year-old male with no significant past medical history presented to the emergency department (ED) with pain, redness, and swelling of his right ankle’s medial aspect for five days. He first noticed his symptoms while mowing grass. He denied any trauma. At the time of symptom onset, he was under home arrest and was wearing an electronic monitoring device on his right ankle. He was concerned that this device might have been contributing to his symptoms and had it moved to his left lower extremity two days before his presentation to the ED. Despite this, his pain, redness, and swelling continued to worsen. Ibuprofen and heating pads improved his pain but did not reduce the swelling. He noted that his pain was aggravated by ambulation, but he maintained the ability to ambulate independently. He denied associated fever or rash. At the time of his presentation to the ED, his pain severity was 7–8 on a 1–10 scale.

**Image 1:**
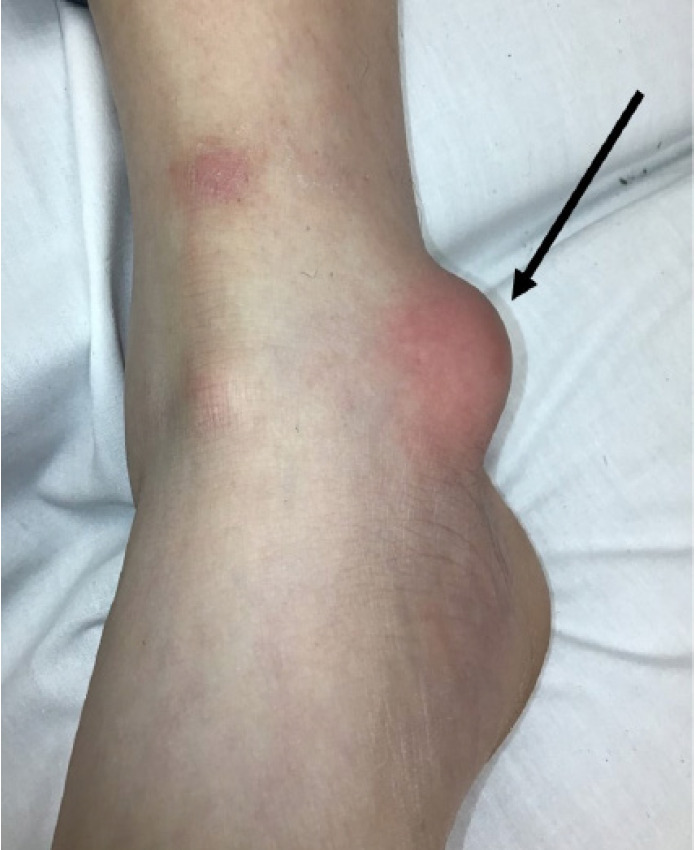
Localized swelling and erythema over the medial malleolus (arrow).

On examination, the patient was well appearing. Initial vital signs were as follows: temperature 36.8°C (98.2°F); heart rate 99 beats per minute; respiratory rate 18 breaths per minute; blood pressure 128/82 millimeters mercury; and pulse oximetry 96% on room air. Erythema and swelling were noted overlying the right medial malleolus (Image 1). The area was tender and fluctuant. There was no evidence of trauma to the skin. No crepitus or drainage was present. Distal sensation and pulses were intact, and his ankle had a full range of motion.

**Image 2:**
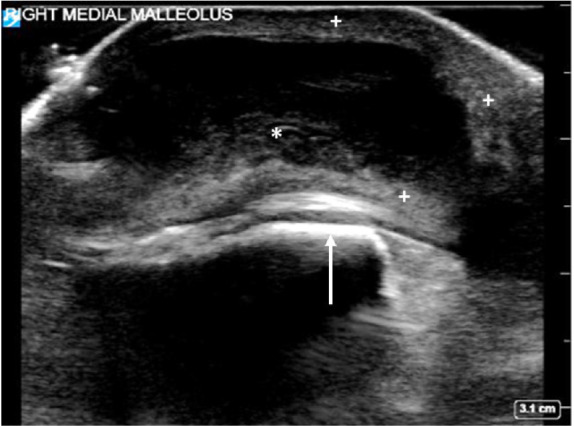
Ultrasound demonstrating a thick-walled (plus signs) bursa containing complex fluid (asterisk), which overlies the medial malleolus (arrow).

Plain radiographs demonstrated marked soft tissue swelling around the ankle joint, most prominent at the medial malleolus. A point-of-care ultrasound was performed, which revealed a 3 centimeter × 1 centimeter thick-walled cystic mass containing fluid of mixed echogenicity, consistent with bursitis (Image 2).

Aspiration of the bursa was performed with an 18-gauge needle, and 3 milliliters of cloudy yellow fluid was removed and sent for culture. No additional studies were obtained. The patient noted decreased pain after the procedure. A presumptive diagnosis of septic medial malleolar bursitis was made, and the patient was discharged with a 10-day course of trimethoprim-sulfamethoxazole. He did not receive antibiotics in the ED.

Gram stain of the aspirated fluid was negative, but culture grew *Staphylococcus aureus*. On telephone follow-up one week later, the patient noted significant symptomatic improvement. Unfortunately, he had not begun taking his antibiotics. He was encouraged to do so. The patient was lost to further follow-up.

**CPC-EM Capsule**

What do we already know about this clinical entity?

Malleolar bursitis is a well described but uncommon cause of acute ankle pain and swelling, often associated with occupational or recreational activities.

What makes this presentation of disease reportable?

Ankle bursitis associated with the use of an electronic monitoring device has not previously been described in the medical literature.

What is the major learning point?

Malleolar bursitis may be associated with the use of an electronic monitoring device. Ultrasound may assist in diagnosis.

How might this improve emergency medicine practice?

Clinicians should include malleolar bursitis in the differential diagnosis of ankle pain and swelling in patients wearing an ankle device.

## DISCUSSION

Bursae are fluid-containing, extra-articular closed sacs that provide cushioning and relieve friction between skeletal and soft tissue structures, including bone-tendon, bone-skin, and tendon-ligament interfaces. Bursae may be classified as deep or superficial. Deep bursae (eg, subacromial, iliopsoas, and retrocalcaneal) form during embryonic development. Superficial bursae reside in the subcutaneous tissue and tend to develop after birth. Examples include the olecranon and prepatellar bursae. Adventitious superficial bursae are acquired bursae that develop in response to repeated trauma, pressure, or friction.^1^,^2^ They lack a proper synovial lining and are variably present. Two such adventitious bursae may be found in the ankle: the medial and lateral malleolar bursae. They reside in the subcutaneous tissues overlying the bony prominences of the medial and lateral malleoli, allowing the overlying skin to glide more easily.^3^–^6^

Bursitis is the inflammation of a bursa characterized by bursa wall thickening and excess bursal fluid accumulation, producing localized pain and swelling. It may be septic or aseptic. Bursitis in some anatomic locations is associated with various occupational and recreational activities, giving rise to numerous descriptive terms such as “carpet layer’s knee” (prepatellar bursitis) and “student’s elbow” (olecranon bursitis).^2^

Aseptic bursitis is commonly caused by repetitive trauma, compression, or shear forces. Still, it may also be caused by crystal deposition or underlying autoimmune disorders such as rheumatoid arthritis or systemic lupus erythematosus.

Septic bursitis occurs when a bursa becomes inoculated with bacteria. This most commonly results from direct penetration of skin flora in the setting of local cutaneous trauma or from the direct spread of an overlying skin infection. Hematogenous spread of infection to bursa has been described but is not common.^1^,^2^ Given the pathogenesis of local inoculation with skin bacteria, it is not surprising that septic bursitis usually occurs in superficial bursae. Patients present with localized pain, swelling, erythema, and warmth. The presence of fever is inconsistent.^2^,^7^

Clinical features may be unreliable in distinguishing septic from aseptic bursitis; definitive diagnosis is established by culture of bursal aspirate. *S aureus* is the causative agent in approximately 80% of cases.^1^,^2^,^7^ Streptococci are the second most common cause. Bursa infections with various other bacterial species, including Gram-negative bacilli and anaerobes, have been described but are rare. The treatment of septic bursitis is variable, and there are no well-established guidelines.^1^ Treatment typically includes a combination of oral or parenteral antibiotics and needle aspiration or incisional drainage, depending upon disease severity and patient risk factors.

Ultrasound can be useful in establishing the diagnosis of bursitis, as demonstrated by our case. Normal bursae are challenging to visualize with ultrasound and are often invisible. In bursitis, the bursa appears as a cystic structure with a thickened hyperechoic wall. The fluid within the bursa may be anechoic, or it may demonstrate mixed echogenicity in the setting of a complex or bloody bursal effusion.^8^ The use of ED point-of-care ultrasonography for bursitis has been described, but its performance and utility have not been well characterized.^9^–^11^

There are approximately 150 bursae in the human body, but the vast majority of bursitis cases encountered by the emergency physician occur in only two: the olecranon or prepatellar bursae.^2^,^4^ Bursitis of the malleolar bursae, as described in our case, is relatively uncommon but deserves inclusion in the differential diagnosis of ankle pain and swelling. Like bursitis in other anatomic locations, malleolar bursitis may be septic or aseptic and is often caused by local microtrauma, shear forces, and compression. Lateral malleolar bursitis has been described in patients who sit cross-legged for prolonged periods of time, including children, tailors, and coal miners working in low-seam underground mine environments.^3^,^6^,^11^,^12^ An association with ill-fitting shoes has also been reported.^12^

Medial malleolar bursitis has been described in figure skaters and attributed to excessive contact pressure and shear forces that occur between the medial malleolus and snuggly fitting skater’s boots while the skater is performing mechanical movements that include jumping, twisting, and changes in direction.^4^,^13^ Aseptic malleolar bursitis often responds well to conservative therapy, including modification of inciting factors such as footwear or activity. Aspiration of the bursa and injection of steroids may be considered.^4^ Surgical bursectomy has been employed for recalcitrant cases. Septic malleolar bursitis is treated with drainage and anti-staphylococcal antibiotics in a manner similar to septic bursitis in other anatomic locations. Surgical intervention may be necessary in some cases.^4^,^5^,^13^

We report a patient with medial malleolar bursitis who had no apparent occupational or recreational risk factor and no underlying medical conditions. He reported having a court-ordered ankle electronic monitoring device on his ankle prior to developing symptoms. To our knowledge, no case of malleolar bursitis in a patient with an electronic monitoring device has been reported in the medical literature. An exhaustive search of the literature failed to reveal any reports of adverse medical effects of ankle electronic monitoring devices; however, there have been recent reports of lower extremity skin irritation and infection on advocacy websites and in the lay press.^14^,^15^ Although it is impossible to definitively establish causation, it is conceivable that such a device may have produced shear or pressure forces on the medial malleolus and microtrauma to the overlying skin provoking the development of septic medial malleolar bursitis in our patient.

## CONCLUSION

Emergency physicians regularly see patients with ankle pain and swelling and must consider a broad differential diagnosis. Septic malleolar bursitis is an uncommon but important cause of ankle pain and swelling that requires prompt diagnosis and intervention. Point-of-care ultrasonography may aid in the diagnosis. Additionally, emergency physicians should be aware of potential complications related to electronic monitoring devices.
